# Elevated G-CSF, IL8, and HGF in patients with definite Meniere’s disease may indicate the role of NET formation in triggering autoimmunity and autoinflammation

**DOI:** 10.1038/s41598-022-20774-8

**Published:** 2022-09-29

**Authors:** Jing Zou, Zikai Zhao, Xianmin Song, Guoping Zhang, Hongbin Li, Qing Zhang, Ilmari Pyykkö

**Affiliations:** 1 Department of Otolaryngology-Head and Neck Surgery, Changhai Hospital, Second Military Medical University, Changhai Road #168, Shanghai, 200433 China; 2 Faculty of Information Technology and Communication Sciences, Tampere University, Tampere, Finland; 3 Hearing and Balance Research Unit, Field of Otolaryngology, School of Medicine, Faculty of Medicine and Health Technology, Tampere University, Tampere, Finland

**Keywords:** Immunology, Molecular biology, Neuroscience, Biomarkers, Diseases, Health care, Medical research, Molecular medicine, Neurology

## Abstract

The etiology and mechanism causing Meniere’s disease (MD) are not understood. The present study investigated the possible molecular mechanism of autoimmunity and autoinflammation associated with MD. Thirty-eight patients with definite MD and 39 normal volunteers were recruited, and 48 human cytokines/chemokines were quantified. In patients with MD pure tone audiograms, tympanograms and standard blood tests were performed. The mean hearing loss in the worse ear was 44.1 dB nHL. Compared to the referents, the concentrations of TNFα, IL1α, IL8, CTACK, MIP1α, MIP1β, G-CSF, and HGF in the sera of patients with MD were significantly elevated, while those of TRAIL and PDGFBB were significantly decreased. The area under the receiver operating characteristic curve (AUC) showed that G-CSF, MIP1α, and IL8 were above 0.8 and could be used to diagnose MD (*p* < 0.01), and the AUCs of CTACK and HGF were above 0.7 and acceptable to discriminate the MD group from the control group (*p* < 0.01). The revised AUCs (1 − AUC) of TRAIL and PDGFBB were above 0.7 and could also be used in the diagnosis of MD (*p* < 0.01). The linear regression showed significant correlations between MIP1α and GCSF, between IL2Rα and GCSF, between IL8 and HGF, between MIP1α and IL8, and between SCF and CTACK; there was a marginal linear association between IP10 and MIP1α. Linear regression also showed that there were significant age-related correlations of CTACK and MIG expression in the MD group (*p* < 0.01, ANOVA) but not in the control group. We hypothesize that G-CSF, IL8, and HGF, which are involved in the development of neutrophil extracellular traps (NETs) and through various mechanisms influence the functions of macrophages, lymphocytes, and dendritic cells, among others, are key players in the development of EH and MD and could be useful in elucidating the pathophysiological mechanisms leading to MD. Biomarkers identified in the present study may suggest that both autoimmune and autoinflammatory mechanisms are involved in MD. In the future, it will be valuable to develop a cost-effective method to detect G-CSF, IL8, HGF, CTACK, MIP1α, TRAIL, and PDGFBB in the serum of patient that have diagnostic relevance.

## Introduction

Meniere’s disease (MD) is characterized by episodic vertigo accompanied by fluctuating sensorineural hearing loss, aural fullness, and tinnitus. The condition can be alleviated by dietary and lifestyle modifications, medication, and invasive interventions such as intratympanic steroid injection, intratympanic gentamicin injection, labyrinthectomy among others, but the etiology and mechanism causing MD are not understood^1,2^. Among the treatment modalities, intratympanic dexamethasone was reportedly capable of controlling vertigo in 91% of patients^3^, and in a randomized study, intratympanically administered corticosteroids were as effective as gentamicin to control vertigo^4^.

Brookes reported significantly increased circulating immune complex levels and an increased incidence of autoantibodies in MD, indicating an autoimmune mechanism^5^. Antibodies against bovine inner ear antigens were detected in the sera of MD patients^6^. An association of autoimmunity with familial patterns of MD leads to longer spells of MD^7^. A link between thyroid autoimmunity and MD was also proposed^8,9^. In addition to potential autoimmunity, autoinflammatory mechanisms may also be involved in the development of MD^9–15^. However, the detailed mechanism of eliciting either autoimmunity or autoinflammation involved in MD has not yet been clarified.

The endolymphatic sac (ES) is suggested to operate as an immune organ in situ, initiating the immune reaction in MD. A study on fresh human ES tissue using DNA microarrays and immunohistochemistry demonstrated multiple key elements of both the innate and adaptive immune reactions^16^. A preliminary study demonstrated that the proteins that appeared in the ES luminal fluid of patients with MD were associated with both innate and adaptive immune reactions^17^. Multiple genetic variants associated with innate immunity and immune regulation as well as the formation of inner ear structures and systemic hemostasis derangement were also detected in patients with sporadic MD in an East Asian population^18^. Therefore, both autoimmune and autoinflammatory reactions might be initiated in the endolymphatic sac of MD patients. If this occurs, then screening the cytokine/chemokine profile may provide a clue to understanding the detailed mechanism of the abovementioned immune reactions in MD patients.

The difference between human autoimmune responses and autoinflammatory responses is not distinctly defined and they may occur in the same subject simultaneously. Autoimmune diseases are characterized by the production of specific autoantibodies resulting from the loss of immune tolerance, the recognition of self-antigens and the activation of T cells and B cells, and these lead to damage to multiple organs owing to a dysregulated adaptive immune response. Cytokines, such as TNFα, IL-1β, and IL-6, are involved in processes modulated by their delivery in extracellular vesicles^9,19,20^. In contrast, autoinflammatory diseases are triggered without detectable autoantibodies or specific T cells and are mediated by cytosolic multiprotein complexes, so-called inflammasomes. Activation of the inflammasome leads to the cleavage of pro-interleukin (IL)-1β and the secretion of active IL-1β^21^. In addition, NF-κB and TNFα signaling are also activated in certain autoinflammatory diseases^19^. Mutations in MEFV, the gene encoding pyrin, which acts as an intra-nuclear regulator of transcription of the peptides involved in inflammation, mainly in the innate immune system and is an upstream signal of IL-1, have been defined as variants specific for autoinflammatory diseases and have been detected in MD^15,19,22^. However, many immunological diseases are mixed-pattern conditions that comprise elements of both autoimmunity and autoinflammation^19,23^.

The present study aimed to identify the cytokine profiles of MD and potential association to autoimmune/autoinflammatory mechanism in MD. The cytokine/chemokine profile in the sera of patients with definite MD was analyzed using premium Luminex.

## Patients and methods

### Clinical data

This study was carried out following the Code of Ethics of the World Medical Association (Declaration of Helsinki) for experiments involving humans that were further updated^24^, and the protocol was reviewed and approved by the ethics committee of Shanghai Changhai Hospital (CHEC2020-107). Written informed consent was obtained from all participants. The inclusion criteria were as follows: (1) a diagnosis of definite MD was made according to the criteria of the Barany Society in 2015^25^ and (2) healthy volunteers without any history of chronic disease or complaining of acute disease. The exclusion criteria were patients with probable MD according to the abovementioned diagnostic criteria and patients with any diseases in the central nervous system, autoimmune or autoinflammatory disease and/or acute infection such as acute infection upper respiratory infection. However, MD patients accompanied by hypertension were not excluded. In total, 38 patients with definite MD (male:female = 1:1, aged 27–81, 53.6 ± 14.9 years) who visited author JZ at the Department of Otolaryngology-Head & Neck Surgery, Changhai Hospital, Second Military Medical University, from April to November 2021 and 39 normal volunteers (male:female = 1:1.17, aged 20–66, 36.2 ± 11.7 years) were recruited. Both the mean ages and range of healthy volunteers were younger than that of MD. All patients had audiological tests for pure tone audiograms and tympanograms and standard blood tests for immunoglobulins, transferrin, β2-microglobulin, α1-microglobulin, complement 3 (C3), C4, ceruloplasmin, anti-streptolysin O antibody, erythrocyte sedimentation rate, rheumatoid factor, C-reactive protein, and the total thyroid hormones triiodothyronine (T3) and thyroxine (T4).

### Quantification of cytokines/chemokines

Two milliliters of blood was taken from each subject at 7:00 am before breakfast, and the serum was stored at − 80 °C until analysis. The time between the last attack and taking the blood sample varied from 1 to 7 weeks (mean 2.2 ± SD 1.8). The human cytokines/chemokines CTACK, FGF basic, G-CSF, GM-CSF, GRO-α, IFN-α2, IFN-γ, IL-1α, IL-1β, IL-1Rα, IL-2, IL-2Rα, IL-3, IL-4, IL-5, IL-6, IL-7, IL-8, IL-9, IL-10, IL-12 (p40), IL-12 (p70), IL-13, IL-15, IL-16, IL-17, IL-18, IP-10, LIF, MCP-1, MCP-3, M-CSF, MIF, MIG, MIP-1α, MIP-1β, NGF-β, PDGF-BB, RANTES, SCF, SCGF-β, SDF-1α, TNF-α, TNF-β, and TRAIL were quantified using a Bio-Plex Pro Human Cytokine Screening Panel, 48-plex #12007283 (Bio-Plex Suspension Array System; Bio-Rad Laboratories Inc., Hercules, USA) according to the manufacturer's instructions. The antibody array experiment was performed by Wayen Biotechnology (Shanghai, China) according to their established protocol. In brief, a total of 50 μL of antibody-conjugated beads was added to the assay plate. Then, 50 μL of serum, standards, the blank, and the controls were added to the plate, and the plate was incubated in a dark room at ambient temperature with shaking at 850 rpm for 2 h. After washing 3 times, 50 μL of biotinylated antibody was added to the plate, which was incubated in a dark room at RT with shaking at 850 rpm for 1 h and then washed 3 times again. Subsequently, a total of 50 μL streptavidin–phycoerythrin was added to the plate. The plate was again incubated in a dark room at ambient temperature with shaking at 850 rpm for 30 min and then washed 3 times. Finally, the plate was read using a Bio-Plex MAGPIX Multiplex Reader (Bio-Rad, Hercules, USA). Bio-Plex Manager 6.0 software was used for data acquisition and analysis. We used a randomized double-blind protocol to evaluate these measurements.

### Statistics

SPSS 28.0 software was used for statistical analysis. For the phenotypes of MD, duration of the disease course, the time interval between the last vertigo attack and the cytokine assay, and both the average thresholds of the speech frequencies (0.5, 1.0, and 2.0 kHz) and that of the most severely impaired frequency (some patients only have hearing loss at one frequency during the early stage) were used in the analysis. For the general immunological phenotypes, C3 and C4 were used in the statistical analysis because only sparse cases demonstrated significant changes for the other tests. The measurements of GM-CSF, IFNα, IL-2, IL-3, IL-4, IL-6, IL-7, IL-10, IL-12(70), IL-12(40), IL-15, MCP-3, βNGF, and VEGF were not applied in the statistical analysis because their signal intensities were out of range of the standard curve. The measurements of eotaxin, GSCF, GROα, HGF, IL1Rα, IL2Rα, IL8, IL13, IL16, IL17, IP10, LIF, MCP1, MIG, MIP1α, MIP1β, PDGFBB, SCGFβ, TNFα, and TRAIL did not have normal distributions and were logarithmically converted to normalize the data. Independent t-tests were performed to compare cytokine/chemokine differences in subjects with MD to the referents, and in MD patients with hypertension to these without hypertension. For variables that showed significant differences in the above independent t-tests, ROC curve classification analysis was performed. Sensitivity, specificity, likelihood ratios, and the AUC of the ROC based on the trapezoidal rule were calculated and judged according to previously reported criteria^26^. Spearman’s rho test was applied to analyze correlations between age and cytokines/chemokines in both MD and referents, between the hearing level of the most severely impaired frequency, and the immunological phenotypes. Linear regression was performed between variables other than internal chemokine/cytokine correlations that showed significant correlations using Spearman’s rho test. The potential cytokine activation that may occur in uniform chain was formulated by exploring the Kendall’s tau between different cytokines among MD patients. *p* < 0.05 indicated statistical significance.

### Ethics approval and consent to participate

The study was approved by the ethics committee of Shanghai Changhai Hospital.

## Results

### Clinical characteristics of MD

Based on the clinical findings, 32 patients had unilateral MD and 6 had bilateral MD. The mean course of MD was 57.8 months (range 1 to 300 months). The mean threshold of hearing at speech frequencies was 44.1 dB nHL with a range of 17 to 88 dB nHL, while that of the most severely impaired frequency, with the majority at 250 Hz, varied from 25 to 115 dB nHL (mean 64.2 ± 25.5). From the standard laboratory tests of C3, C4, T3, transferrin, IgG, IgM, and ceruloplasmin, among others, we observed significant variations from normal laboratory values in a few individuals (Table 1).

**Table 1:** Results of standard laboratory tests in patients with Meniere’s disease.

Items	Cases with various levels			Abnormal rate (%)
	Normal	Low	High	
β2MGB	35	3	0	8
α1MGB	37	0	1	< 5
Transfer	31	6	1	21
IgA	35	1	2	8
IgG	33	1	4	11
IgM	34	4	0	5
IgE	36	0	2	13
C3	27	11	0	29
C4	29	8	1	24
Cerulopl	34	4	0	13
Anti-‘O’	37	0	1	< 5
ESR	32	0	6	16
RF	37	0	1	< 5
CRP	38	0	0	0
T3	32	5	1	16

Low and high values are located outside the 95% confidence limit of normal values.

α1MGB, alpha1-microglobulin; Anti-‘O’, Anti-Streptolysin O antibody; β2MGB, beta2-microglobulin; C3 and C4, complement 3 and 4; Cerulopl, ceruloplasmin; CRP, C-reactive protein; ESR, erythrocyte sedimentation rate; IgA, IgG, IgM, and IgE, immunoglobulins A, G. M, and E; RF, rheumatoid factor; Transfer, transferrin.

### Cytokines/chemokines and internal correlations

**Figure 1:**
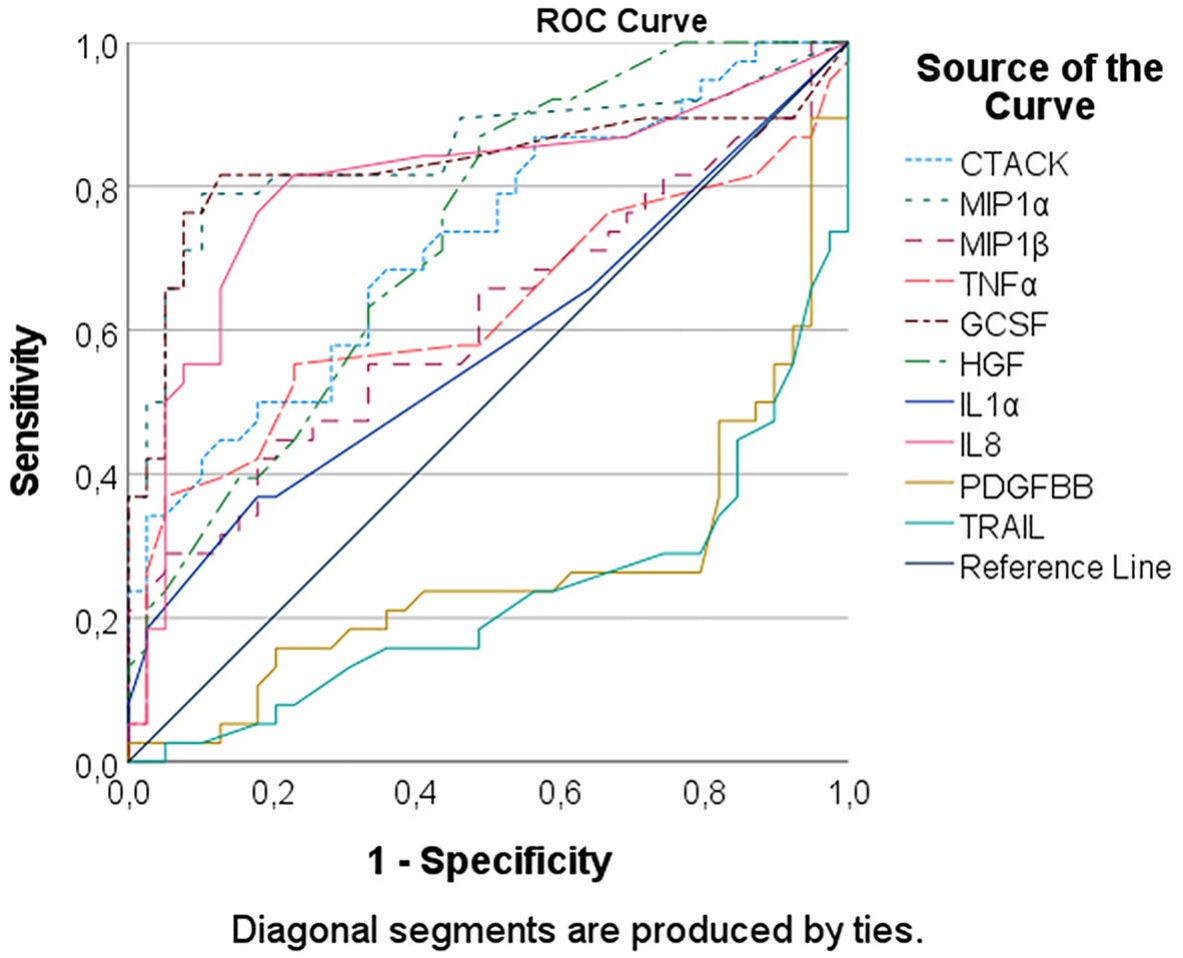
ROC curve of clustering analysis of cytokines contributing in diagnosis of MD.

Compared to the healthy controls, the levels of TNFα, IL1α, IL8, CTACK, MIP1α, MIP1β, G-CSF, and HGF in the sera of patients with MD were significantly elevated, while those of TRAIL and PDGFBB were significantly decreased (independent sample t-test) (Supplementary information 1). In the detailed analysis, the AUC showed that G-CSF, MIP1α, and IL8 were above 0.8 and could be used to discriminate MD from the referents (*p* < 0.01), and the AUCs of CTACK and HGF were above 0.7 and assisted in discriminating the 2 groups (*p* < 0.01). The revised AUCs (1 − AUC) of TRAIL and PDGFBB were above 0.7 and were also useful for discrimination (*p* < 0.01) (Fig. 1; Table 2).

**Table 2:** Area under the ROC curve of clustering analysis of cytokines diagnosing Meniere’s disease.

Area under the curve					
Test result variable(s)	Area	SE^a^	Asymptotic Sig.^b^	Asymptotic 95% confidence interval	
				Lower bound	Upper bound
CTACK	.723	.057	.001	.611	.836
MIP1α	.848	.048	.000	.754	.941
MIP1β	.615	.065	.083	.487	.742
TNFα	.625	.066	.059	.495	.755
GCSF	.829	.053	.000	.725	.933
HGF	.731	.056	.000	.621	.842
IL1α	.576	.066	.250	.447	.706
IL8	.801	.054	.000	.695	.908
PDGFBB	.263	.060	.000	.146	.380
TRAIL	.227	.054	.000	.121	.334

The test result variable(s): CTACK, TransfMIP1α, TransfMIP1β, TransfTNFα, TransfGCSF, TransHGF, TransIL1α, TransIL8, TransfPDGFBB, TransfTRAIL has at least one tie between the positive actual state group and the negative actual state group. Statistics may be biased.

^a^Under the nonparametric assumption.

^b^Null hypothesis: true area = 0.5.

**Figure 2:**
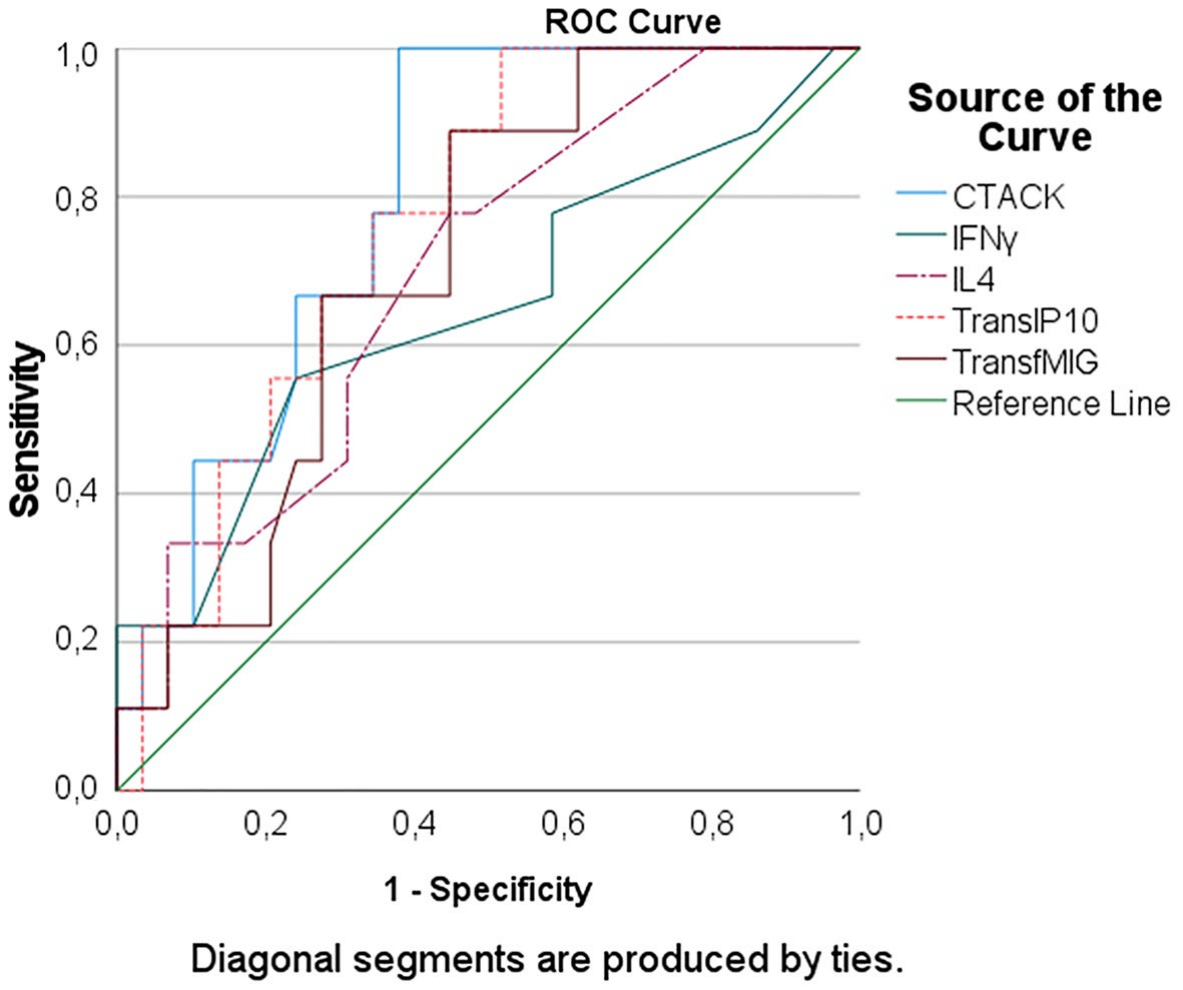
ROC curve of clustering analysis of cytokines that may distinguish MD patients with hypertension from these without hypertension.

Within MD group, patients with hypertension had significantly higher levels of CTACK, IP10, MIG, IFNγ, and IL4 than these without hypertension (independent sample t-test) (Supplementary information 2). The AUC of CTACK, IP10, and MIG were above 0.7 (Fig. 2; Table 3).

**Table 3:** Area under the ROC curve of clustering analysis of cytokines diagnosing Meniere’s disease with hypertension.

Area under the curve					
Test result variable(s)	Area	SE^a^	Asymptotic Sig.^b^	Asymptotic 95% confidence interval	
				Lower bound	Upper bound
CTACK	.799	.072	.007	.657	.940
IFNγ	.649	.114	.181	.426	.873
IL4	.697	.094	.077	.513	.882
IP10	.762	.079	.019	.607	.918
MIG	.715	.087	.055	.545	.884

The test result variable(s): CTACK, IFNγ, IL4, TransfMIG has at least one tie between the positive actual state group and the negative actual state group. Statistics may be biased.

^a^Under the nonparametric assumption.

^b^Null hypothesis: true area = 0.5.

**Figure 3:**
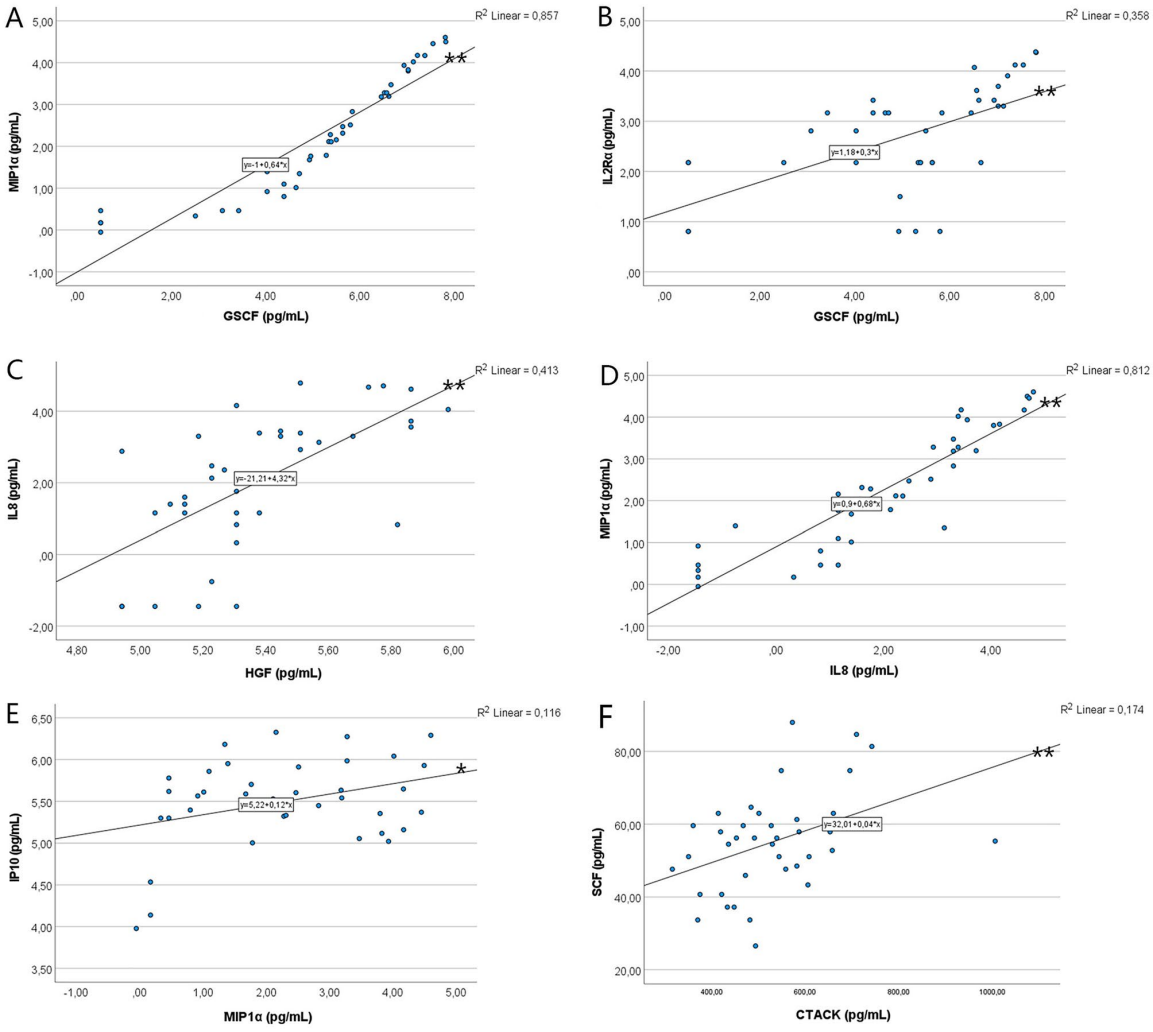
Correlations between chemokines in MD as demonstrated by linear regression. *p < 0.05; **p < 0.01 (ANOVA).

**Figure 4:**
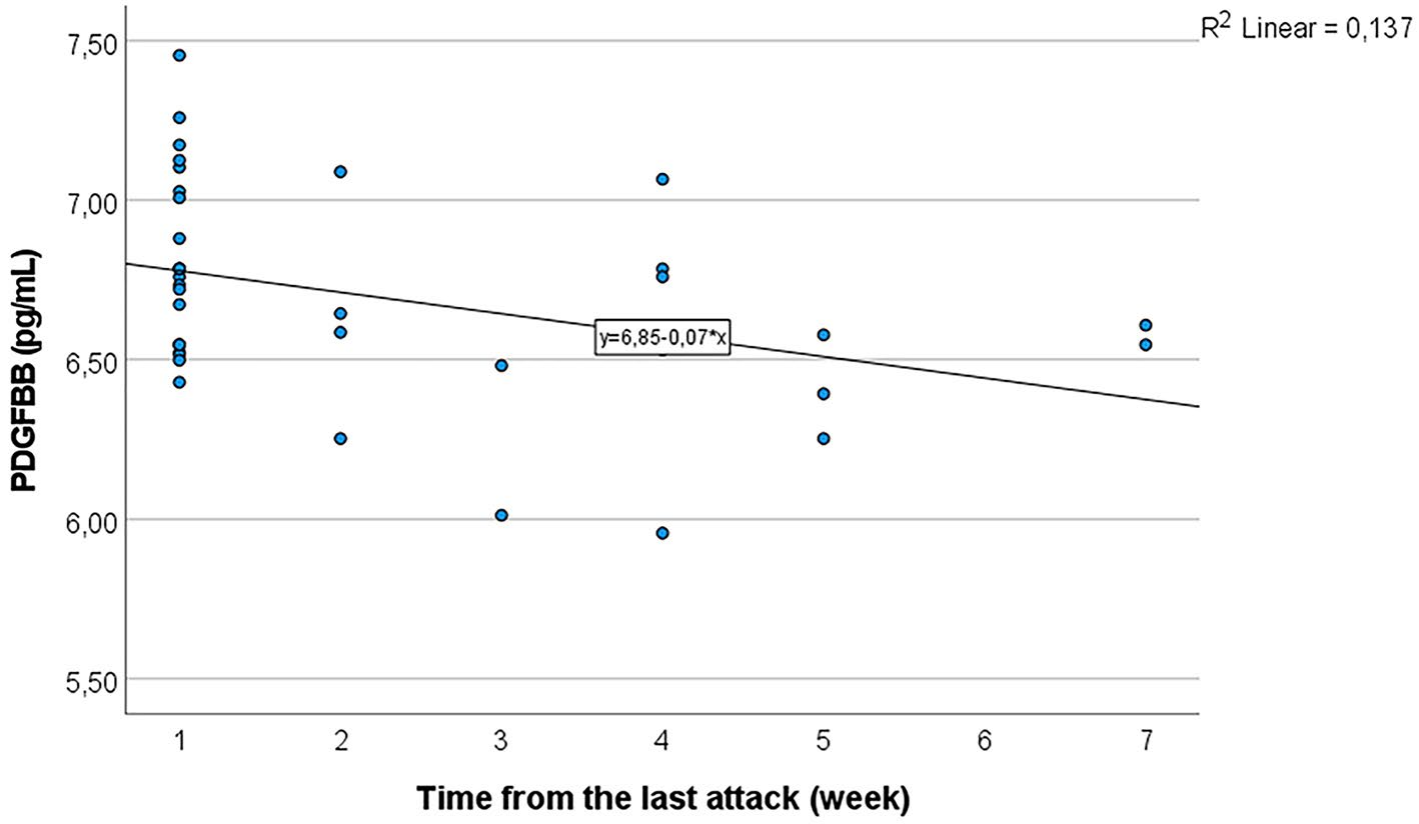
Correlations between the time of the last attack and PDGFBB in MD as demonstrated by linear regression. *p* < 0.05 (ANOVA).

**Figure 5:**
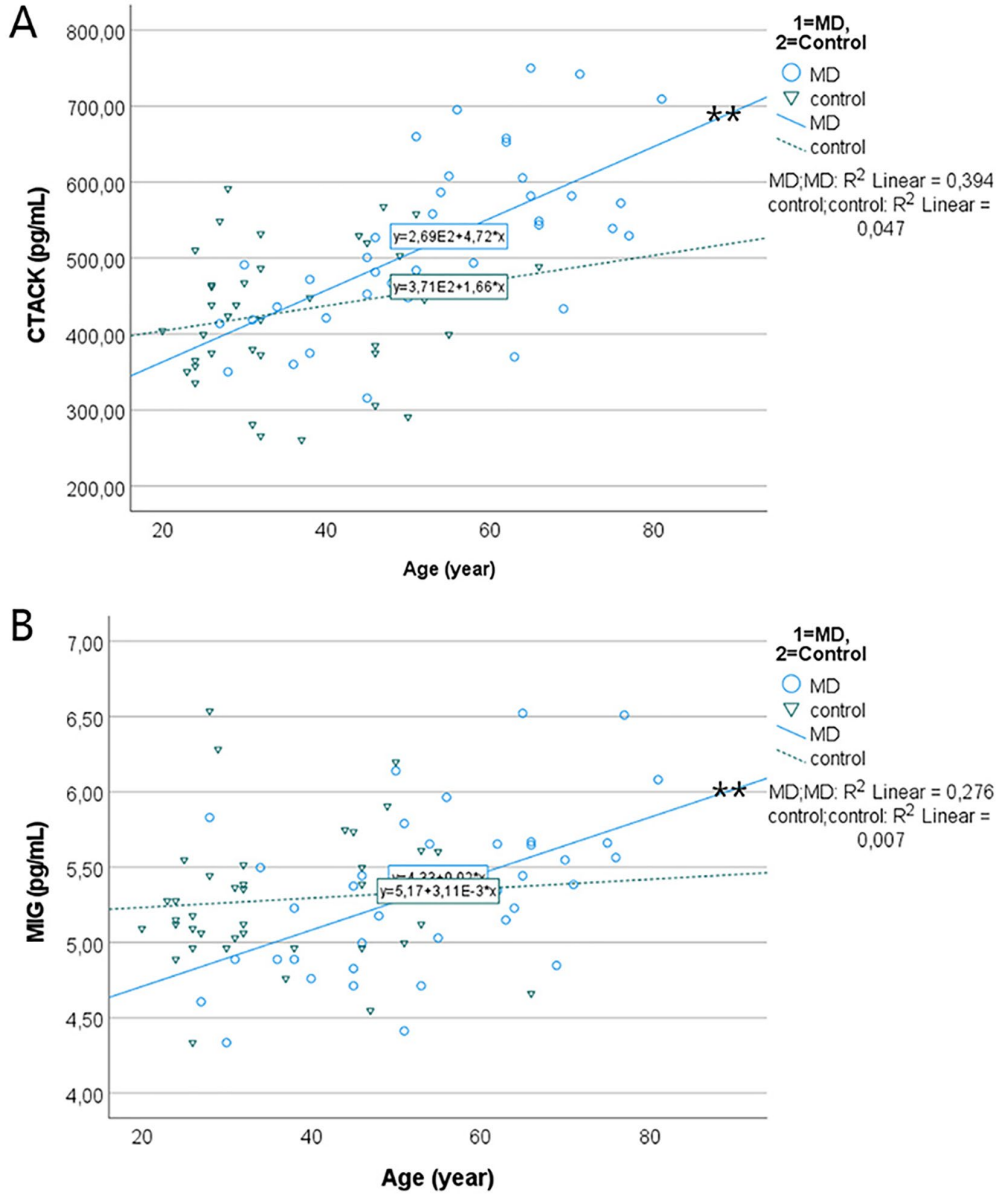
Correlations between age and CTACK and MIG demonstrated by linear regressions. **p < 0.01 (ANOVA).

Internal correlations were further analyzed using linear regression for variables that showed significant correlations by Spearman’s rho test (Supplementary information 3). The linear regression showed significant correlations between MIP1α and GCSF (R^2^ = 0.857, *p* < 0.001), between IL2Rα and GCSF (R^2^ = 0.358, *p* < 0.001), between IL8 and HGF (R^2^ = 0.413, *p* < 0.001), between MIP1α and IL8 (R^2^ = 0.812, *p* < 0.001), and between SCF and CTACK (R^2^ = 0.174, *p* < 0.01); there was a marginal linear association between IP10 and MIP1α (R^2^ = 0.116, *p* = 0.037) (Fig. 3). Linear regression also showed that there were significant negative correlations between the time of the last attack and PDGFBB (Fig. 4), and positive age-related correlations of CTACK (R^2^ = 0.394, *p* < 0.001) and MIG (R^2^ = 0.276, *p* < 0.001) expression in the MD group (*p* < 0.01, ANOVA) but not in the control group (Fig. 5).

We searched the association between different cytokines by exploring the Kendall’s tau (Supplementary information 4) between different cytokines to formulate which of the cytokine activation may occur in uniform chain. Thus, we grouped those with correlation coefficient greater than 0.7 in one group and those between 0.5 to 0.7 in another group as these could be indicate separate activation pattern. The cytokines IL8, MIP1α and GCSF had correlation greater than 0.7. In addition, the GSCF also activates TNFα. The cytokines IL1α, IL1β, IL16, bFGF, IL2Rα, IL4, LIF, and TNFα could form one additional activation branch. IL4, Eotaxin, IL17, and LIF form another branch.

## Discussion

### Potent NET-associated activity indicated by elevated serum levels of chemokines in MD patients

**Figure 6:**
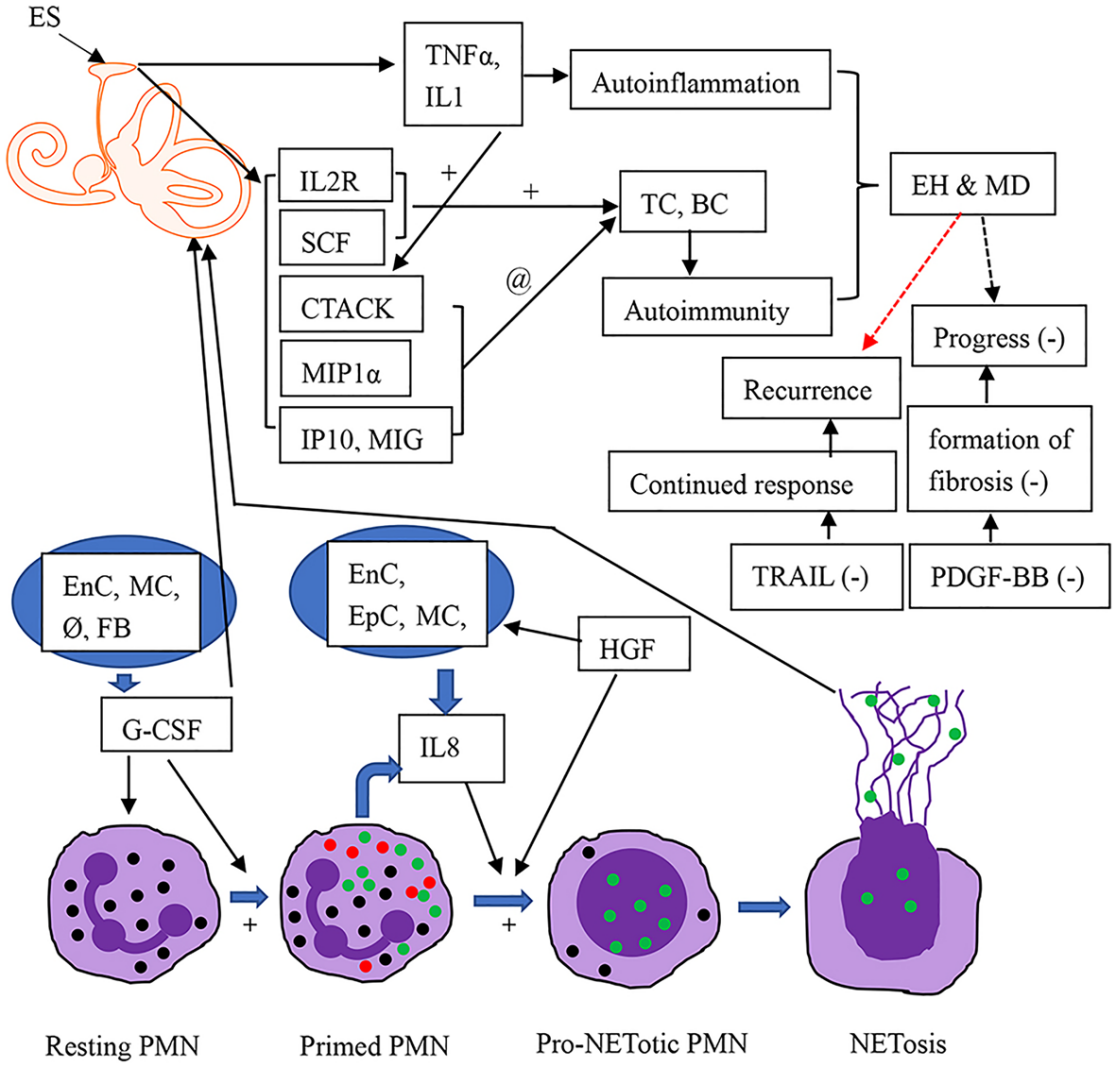
Involvement of NET formation in initiating the pathological process of MD. BC: B cell; EH: endolymphatic hydrops; ES: endolymphatic sac; EnC: endothelial cells; EpC: epithelial cells; FB: fibroblasts; MC: monocytes; PMN: polymorphonuclear neutrophil; Ø: macrophages. Green dots: key components of the NETotic cascade, such as protein arginine deiminase-4, neutrophil elastase, and myeloperoxidase; red dots: proinflammatory cytokines, such as IL8 and TNFα; TC: T cell. “+”: promotion; “−”: downregulation; “@”: recruitment or homing. Dashed arrow: adjustment of the MD course.

We observed that various cytokines participating in autoimmunity and autoinflammation were up- and downregulated in MD. The AUC analysis showed that G-CSF, IL8, HGF, CTACK, MIP1α, TRAIL, and PDGFBB have diagnostic relevance, suggesting that these chemokines play an important role in MD. IL8, MIP1a and GCSF also had internal correlation greater than 0.7 indicting that they probably act in the same pathway and are activated by the same mechanisms. The G-CSF also correlated highly significantly with TNF-α indicating that the outcome of this pathway is harmful for inner ear. It was reported that G-CSF, IL8, and HGF are involved in the development of NETs through a mechanism by which G-CSF stimulates the production and release of neutrophils and primes neutrophil programming toward NET generation. HGF provokes neutrophils to release IL8, and IL8 together with HGF activate CXCR1 and CXCR2 to induce the formation of NETs^27–30^. A similar release of NETs from neutrophils has been identified as a mechanism used for bacterial killing^31^. NETs are network structures comprised of chromosomal DNA, granule proteins (myeloperoxidase, lactoferrin, neutrophil elastase, high mobility group box 1, cathepsin G, proteinase 3, and leucine-leucine-37), and citrullinated histone H3 that are catalyzed by PAD4 and released by neutrophils upon activation. Cytokine cascades of NETs are also involved in noninfectious diseases with the mechanisms of autoimmunity and autoinflammation^32,33^. Therefore, we hypothesize that NET formation might be involved in initiating the pathological process of MD as consequence of the enhanced production of specific chemokines (Fig. 6).

TRAIL is a member of the TNF superfamily, and is involved in the apoptotic removal of senescent, chronically activated, or stressed immune cells at sites of inflammation to regulate innate and adaptive immune responses by terminating the response and by limiting thereby tissue damage and the risk of autoimmunity^34^. Downregulation of TRAIL in MD patients may fail to sufficiently resolve the inflammation and causes the inflammatory impairment to enter a chronic course. PDGF‑BB has been reported to recruit mesenchymal cells, such as pericytes and smooth muscle cells, and induce their differentiation into fibroblasts to form fibrosis^35^. Downregulated PDGF-BB, and negative correlations between the time of the last attack and PDGFBB in MD patients in the current study might indicate that there is a mechanism in the body trying to prevent the formation of fibrosis.

### Existence of molecular machinery in the inner ear and ES to form NETs

It has not yet been confirmed whether NET formation induced by these chemokines exists in the inner ear. It was recently reported that G-CSF is necessary for neutrophil recruitment in the cochlea of C57BL/6 mice following intravenous injection of lipopolysaccharide solution^36^. In DFNB39 mice, the associated nonsyndromic hearing loss resulted from noncoding mutations that may influence HGF-induced signaling^37^. There were significant correlations between the concentration of IL8 and that of G-CSF, HGF, and MIP1α, indicating the existence of an intrinsic link among them in MD. Neutrophilic infiltration to the ES or inner ear is necessary to form NETs. It was previously reported that osmotic challenge of the inner ear activated the periaqueductal bone marrow and induced chemotactic attraction of monocytes, neutrophils and eosinophilic leukocytes to the ES^38^. The detailed mechanism of NET formation remains unclear but it may be caused by chromosomal DNA release following histone depolymerization involving myeloperoxidase, neutrophil elastase, and peptidylarginine deiminase 4^39^. Interestingly, one of the MD patients in the current study carried multiple genetic variants associated with both autoimmune and autoinflammatory reactions and demonstrated vascular congestion over the ES, indicating vasculitis^15^.

### Cytokines potentially engaged in autoimmune reaction of MD

Linear regression demonstrated significant correlations between MIP1α and GCSF, between IL2Rα and GCSF, and between MIP1α and IL8. These results are likely to indicate that MIP1α and IL2Rα might interact with GCSF signaling and that MIP1α might also interact with IL8 signaling in MD. MIP1α and MIP1β are C-C chemokines and exhibit different chemoattractant potentials; human MIP1β tends to attract CD4^+^ T lymphocytes, while human MIP1α appears to be a more potent chemoattractant with a broader specificity, attracting B cells and cytotoxic T cells as well as CD4^+^ T cells^40^. In human monocytes, MIP-1α expression might be induced by costimulation with palmitate and TNFα involving the TLR4-IRF3 pathway and then be amplified by oxidative stress^41^. The expression of MIP-1α in human ES fibroblasts was upregulated upon polyinosinic-polycytidylic acid stimulation through the TLR3 mechanism^42^. IL-2 plays a role through binding to IL2R in the maintenance of T_Reg_ cells and the differentiation of CD4^+^ and CD8^+^ T cells into effector T cells and CD8^+^ T cells into memory cells. IL-2Rα (also known as CD25) is the third chain of the trimeric IL-2R and it functions to increase the affinity of IL-2R for its ligand^43^.

Linear regression also demonstrated that there was a marginal linear correlation between IP10 and MIP1α, indicating that IP10 may interact with MIP1α signaling. The concentrations of MIG were age-related in the MD group but not in the control group. Both IP-10 and MIG are ligands of the CXCR3 receptor that are expressed in Th1 cells, B cells, NK cells, dendritic cells, activated T lymphocytes, epithelial cells, and endothelial cells. CXCR3 signaling is involved in the recruitment of activated T cells and the development of autoimmune diseases^44–50^.

The concentrations of CTACK were age-related in the MD group but not in the control group, and linear regression showed a significant correlation between SCF and CTACK. CTACK/CCL27 is another C-C chemokine that was originally detected in the mouse epidermis and keratinocyte of human skin and it selectively chemoattracts CLA^+^ memory T cells^51^. CTACK plays a critical role in skin allergic processes, including atopic dermatitis and delayed-type hypersensitivity reactions^52,53^. TNFα and IL1β induce CCL27 production, whereas glucocorticosteroid suppresses it^52^. CTACK seems to be involved in several disease processes, such as in *multiple sclerosis* patients (appearing in the sera and cerebral spinal fluids)^54,55^ and in *chronic obstructive pulmonary* disease patients (in the sera)^56^. Higher serum levels of CTACK together with other cytokines in *multiple sclerosis* patients were associated with more severe disabilities than mild forms^57^. Human ES luminal fluid contains keratin types I and II and other skin-specific proteins^58^, which may come from inner ear epithelium facing the endolymph. CTACK may also be produced in the human inner ear in a mechanism similar to that in the skin^59^. The present study also showed that higher levels of CTACK, IP-10, and MIG were associated to comorbidity of MD and hypertension, and there were positive age-related correlations of CTACK in the MD group but not in the control group. These results are likely to indicate that both C-C and CXCR3 chemokine signaling induced autoimmune reaction may play a role in the more severe disease among aged persons along with the progressing of MD that was found in *multiple sclerosis*^44–50,57^. SCF is the principal growth factor of mast cells and is secreted by fibroblasts, stromal cells, and endothelial cells. Through binding to the c-Kit receptor, SCF-induced signaling mediates inflammatory and allergic diseases with the autoimmune mechanism^60,61^.

### Cytokines potentially engaged in autoinflammation of MD

Kendall’s tau analysis showed that the cytokines IL1α, IL16, bFGF, ILR2α, IL4, LIF, IL1β, and TNFα could form one additional activation branch. *Familial Mediterranean fever* is an autoinflammatory disease in which large amounts of IL-1β-bearing NETs are released during attacks, and the NETs further amplify IL-1β production by peripheral blood mononuclear cells^62^. It is well known that both IL1α and IL1β activate the same receptor and induce similar biological effects, but IL1α is present in healthy cells in a wide variety of cells and it is expressed in hematopoietic and nonhematopoietic cells^63^. TNF receptor activation was also indicated in the development of autoinflammatory disease^64,65^.

There is an obvious limitation in the study, which is that NET formation in MD was not directly proven, although it was indicated by elevated chemokines. Nine patients with MD had hypertension under treatment. The hypertension could alter the cytokine profile as we observed that these 9 patients differed from other MDs by CTACK, IP10, MIG, IFNγ, and IL4 levels. However, CTACK, IP10, MIG, and were reportedly associated with age instead of hypertension^66^. In the present study, GCSF, IL8, HGF, MIP1α, TRAIL, and PDGFBB in patients with MD were not influenced by hypertension. Therefore, hypertension does not contribute to the mechanism of potential NET formation in MD. Hypertension seems to influence the vertigo attacks and quality of life^67^. Thus, hypertension may be regarded mainly as contributory factors affecting the complaint pattern but so far very few epidemiological studies have been carried out in this aspect. As the hypertension is not considered to be etiological factor in MD, we did not remove these patients. But this observation warrants a further study to control hypertension as a possible confounder in cytokine analysis. This study was carried out in the non-ictal phase of the disease. It would be important to know which cytokines are involved in the ictal phase of MD. This would help us to further explore the pathophysiological mechanisms of MD.

## Conclusion

The current study demonstrated that G-CSF, IL8, HGF, CTACK, and MIP1α have diagnostic relevance for MD. G-CSF, IL8, and HGF are involved in the development of NETs and influence the functions of macrophages, lymphocytes, and dendritic cells, among others, through various mechanisms involved in MD. Biomarkers identified in the present study may suggest that both autoimmune and autoinflammatory mechanisms are involved in MD. In the future, it will be valuable to develop a cost-effective method to detect G-CSF, IL8, HGF, CTACK, MIP1α, TRAIL, and PDGFBB in the serum of patient that have diagnostic relevance.

## Supplementary Information

Supplementary Information 1.

Supplementary Information 2.

Supplementary Information 3.

Supplementary Information 4.

## Data Availability

All data generated or analyzed during this study are included in this published article.

## Abbreviations

AUC: Area under the curve
CTACK: Cutaneous T-cell attracting chemokine
CLA: Cutaneous lymphocyte-associated antigen
ES: Endolymphatic sac
bFGF: Basic fibroblast growth factor
G-CSF: Granulocyte colony-stimulating factor
Gd-DTPA: Gadolinium-diethylenetriamine pentaacetic acid
GM-CSF: Granulocyte-macrophage colony-stimulating factor
GRO-α: Growth related oncogene-α
HGF: Hepatocyte growth factor
IFN-α2, IFN-γ: Interferon-α2, -γ
IL-1α, IL-1β, IL-1Rα, IL-2, IL-2Rα, IL-3, IL-4, IL-5, IL-6, IL-7, IL-8, IL-9, IL-10, IL-12 (p40), IL-12 (p70), IL-13, IL-15, IL-16, IL-17, IL-18: Interlukin-1α, -1β, -1 receptor-α, -2, -2 receptor-α, -3, -4, -5, -6, -7, -8, -9, -10, -12-β chain, 12 heterodimer of α and β chains, -13, -15, -16, -17, -18
IP-10: IFN-induced chemokine protein 10
LIF: Leukemia inhibitory factor
MCP-1, MCP-3: Monocyte chemoattractant protein-1, -3
M-CSF: Macrophage colony-stimulating factor
MD: Meniere’s disease
MEFV: Mediterranean fever
MIF: Macrophage migration inhibitory factor
MIG: Monokine induced by IFN-γ
MIP-1α, MIP-1β: Macrophage inflammatory protein-1α, -1β
NF-κB: Nuclear factor-κB
NGF-β: Nerve growth factor-β
nHL: Normalized hearing level
NK: Natural killer
NLRP3: Nod-like receptor protein 3
PAD4: Protein arginine deiminase-4
PDGF-BB: Platelet-derived growth factor-BB
RANTES: Regulated on activation, normal T-cell expressed and secreted
ROC: Receiver operating characteristic
SCF: Stem cell factor
SCGF-β: Stem cell growth factor-β
SDF-1α: Stromal cell-derived factor-1α
TNF-α, TNF-β: Tumor necrosis factor-α, -β
TRAIL: TNF-related apoptosis-inducing ligand
T_Reg_: Regulatory T
VEGF: Vascular endothelial growth factor
